# Infection of *Nigrospora nonsegmented RNA Virus 1* Has Important Biological Impacts on a Fungal Host

**DOI:** 10.3390/v14040795

**Published:** 2022-04-12

**Authors:** Xiaoyi Wang, Jialiang Lai, Honghao Hu, Jinrui Yang, Kai Zang, Feiyan Zhao, Guohong Zeng, Qiansheng Liao, Zhouhang Gu, Zhiyou Du

**Affiliations:** College of Life Sciences and Medicine, Zhejiang Sci-Tech University, Hangzhou 310018, China; 202020801069@mails.zstu.edu.cn (X.W.); 201920201018@mails.zstu.edu.cn (J.L.); 201920201043@mails.zstu.edu.cn (H.H.); cmvzstu@gmail.com (J.Y.); 51201300103@stu.ecnu.edu.cn (K.Z.); 51201300102@stu.ecnu.edu.cn (F.Z.); zgh20180105@zstu.edu.cn (G.Z.); qshliao@yahoo.com (Q.L.)

**Keywords:** mycovirus, *Nigrospora nonsegmented RNA virus 1*, *Nigrospora oryzae*, pigmentation, pathogenicity, hypovirulence, thermotolerance

## Abstract

*Nigrospora nonsegmented RNA virus 1* (NoNRV1) has been reported previously in the fungus *Nigrospora oryzae*, but its biological effects on its host are unknown. In this work, we isolated a strain 9-1 of *N. oryzae* from a chrysanthemum leaf and identified NoNRV1 infection in the isolated strain. The genome sequence of NoNRV1 identified here is highly homologous to that of the isolate HN-21 of NoNRV1 previously reported; thus, we tentatively designated the newly identified NoNRV1 as NoNRV1-ZJ. Drug treatment with Ribavirin successfully removed NoNRV1-ZJ from the strain 9-1, which provided us with an ideal control to determine the biological impacts of NoNRV1 infection on host fungi. By comparing the virus-carrying (9-1) and virus-cured (9-1C) strains, our results indicated that infection with NoNRV1 promoted the pigmentation of the host cells, while it had no discernable effects on host growth on potato dextrose agar plates when subjected to osmotic or oxidative stress. Interestingly, we observed inhibitory impacts of virus infection on the thermotolerance of *N. oryzae* and the pathogenicity of the host fungus in cotton leaves. Collectively, our work provides clear evidence of the biological relevance of NoNRV1 infection in *N. oryzae*, including pigmentation, hypovirulence, and thermotolerance.

## 1. Introduction

Mycoviruses, also known as fungal viruses, are widespread in major fungal species, including mushroom, yeasts, oomycetes, and filamentous fungi. The majority of mycoviruses have a genome composed of one or more double-stranded RNA (dsRNA) segments [1]. Recently, an increasing number of mycoviruses with single-stranded RNA genomes have been reported [2,3,4,5,6,7,8,9]. In addition, a plant geminivirus-related DNA mycovirus was also reported [10]. Mycoviruses, such as yeast LA and M viruses, usually do not cause obvious viral symptoms [11,12], which is consistent with the limited influence of mycoviral infection on gene expression in their fungal hosts [13,14,15]. In some cases, certain mycoviruses induce dramatic changes in their hosts, including irregular growth, abnormal pigmentation, and altered virulence [11,12,16]. The ability of mycoviruses to reduce the virulence of fungal hosts, known as hypovirulence, has been proved to be a powerful strategy to control fungal diseases in agriculture [17]. One of the well-known hypoviruses is *Cryphonectria hypovirus 1* (CHV1), which causes hypovirulence in chestnut blight fungus *Cryphonectria parasitica* and has been used as a biocontrol agent [1,2,18,19]. In some cases, mycoviruses are even obligate partners in mutualistic relationships with fungal hosts. For instance, *Curvularia thermal tolerance virus* (CThTV)-infected *C. protuberata* is required for the growth of a panic grass, *Dichanthelium lanuginosum*, in soils with temperatures >50 °C [20]. Transcriptomic analyses of fungi with or without infection of this virus suggest that CThTV infection promotes melanin biosynthesis [21], which was reported to be associated with fungal thermotolerance [22,23].

Biological pigments are present ubiquitously in nature and have complicated chemical compositions and variable colorization properties. If not all, many fungi produce pigments which serve various biological functions, including harnessing solar energy for metabolic use and protection against ionizing radiation [24]. Some mycoviruses have been revealed to modulate host pigmentation [25,26,27]. CHV1-EP713 is a well-studied hypovirus that is associated with reduced orange pigmentation in its host *C. parasitica* [28]. The brown-black pigments generated from microorganisms have been referred to as ‘melanin’ and ‘melanin-like’. Melanin is a polymer of phenolic compounds with a negative charge and hydrophobicity [29]. Fungi are rich in melanin that is deposited in the inner or outer layers of cell walls depending on the fungal species. Microscopic studies of the overall architecture of the melanin polymer in fungi reveal that melanin forms granular particles in the cell wall [30,31]. Melanin is an important protective factor against adverse environmental stresses, such as UV radiation, drying, and high osmosis [24]. A previous work reported that CThTV upregulates the expression of host scytalone dehydratase—a key enzyme in the melanin biosynthetic pathway [21]—suggesting that CThTV infection might promote melanin accumulation in fungal host cells. This suggestion is supported by recently published work on the same virus [32].

*Nigrospora oryzae* is a widespread endophyte or weak pathogenic fungus that infects a broad variety of crops, including maize (*Zea mays*) [33], rice (*Oryza sativa*) [34], sorghum (*Sorghum* sp.) [35], cotton (*Gossypium hirsutum*) [36], and other plants [37,38,39,40,41,42]. Recently, a genome draft of *N. oryzae* was released [43] which will definitely accelerate basic research on this pathogenic fungus. So far, several mycoviruses have been found in *N. oryzae*, including *N. oryzae victorivirus 1* [44], *N. oryzae victorivirus 2* [45], *N. oryzae mitovirus 1/2* [45], *N. oryzae fusarivirus 1* [46], *N. oryzae partitivirus 1* [47], and *N. oryzae nonsegmented RNA virus 1* (NoNRV1) [48], which would make *N. oryzae* an ideal microorganism for the study of mycovirus–host interactions. NoNRV1 is an unclassified virus and has a dsRNA genome of 2857 bp encoding two separated open reading frames (ORFs). ORF1 encodes a protein of unknown function, and ORF2 is responsible for encoding a putative RNA-dependent RNA polymerase (RdRP) [48]. That is all we know about the virus since the previous report on its genome sequence. In this work, we identified a novel isolate of NoNRV1 from a strain of *N. oryzae* isolated from a chrysanthemum plant, analyzed its genome sequence and phylogenesis, and tested its biological effects on the fungal host. 

## 2. Materials and Methods

### 2.1. Isolation and Identification of N. oryzae

A chrysanthemum leaf with mild mosaic symptoms was collected in 2018 at Xinsha Island, Zhejiang Province, China. *N. oryzae* strain 9-1 was isolated from the surface-disinfected leaf on potato dextrose agar (PDA) plates at 28 °C in darkness. The isolated fungus was maintained on PDA plates, and its hyphal morphology and spore structure were observed using a Nikon light microscopy (Jiangsu, China). To identify the isolated strain, the internal transcribed region (ITS) fragment was amplified and sequenced according to the reported procedure [49]. The obtained ITS sequence was used as a query for a BLAST search of homologous sequences deposited in GenBank.

### 2.2. Extraction and Molecular Cloning of Viral dsRNA

DsRNA fragments were isolated from *N. oryzae* strain 9-1 according to the methodology reported previously with a slight modification [50], CF-11 cellulose being replaced with Sigmacell cellulose (S6790, Sigma, Steinheim, Germany). The molecular cloning of dsRNA was performed according to the previous report [50]. Briefly, the dsRNA fragment was mixed with Primer A (5′-PO4-TCTTCGGGTGTCCTTCCTCG-NH2-3′, Sangon, Shanghai, China), T4 RNA ligase (NEB) and T4 DNA ligase (NEB). The mixture was incubated at 37 °C for 3 hr. The partial duplex was repaired with 2×Taq PCR StarMix (Genstar, Nanjin, China) at 68 °C for 30 min and precipitated by the addition of 2.5 volumes of ethanol with 10% 3M sodium acetate (pH 5.4). Following denaturation of the tailed dsRNA at 99 °C for 3 min and ice incubation for 5 min, first-strand cDNA was synthesized using the complementary Primer B (5′-CGAGGAAGGACACCCGAAGA-3′) and SuperScript^TM^ III reverse transcriptase (Invitrogen, Waltham, MA, USA), according to the manufacturer’s instructions. The full-length cDNA was obtained by a regular PCR using Primer B and Q5 High-Fidelity DNA Polymerase (NEB). The resultant cDNA was cloned into the vector pLB-simple (Tiangen, Beijing, China) for sequencing. 

### 2.3. Ribavirin Treatment of the Isolated Fungus

As a nucleoside analog, the drug Ribavirin was reported to be efficient to cure specific mycoviruses [51]. To remove NoNRV1-ZJ, here we cultured the isolated fungus 9-1 on PDA plates containing 100 mM Ribavirin at 28 °C for 4 days, as described previously [52]. Then, the mycelia were transferred by hyphal-tipping to new Ribavirin-containing PDA plates. After 5 successive passages, NoNRV1 was determined in both untreated and Ribavirin-treated mycelia by RNA gel blot and RT-PCR, as described below. 

### 2.4. RNA Gel Blot

To assess the efficacy of curing NoNRV1-ZJ from its host 9-1 by Ribavirin treatment, both positive and negative strands of the virus were detected by RNA gel blotting assays, as described previously [53]. Briefly, total RNAs were extracted from the untreated (9-1) and drug-treated (9-1C) mycelia using TRIzol (Thermo-Fisher), separated in 1.5% agarose gel containing 7% formaldehyde, and transferred onto a Hybond + nylon membrane (GE). The positive and negative strands of the viral genome were separately probed using the digoxigenin (DIG)-labeled DNA oligos, oligo-S (5′-ACAAGACGCTCATTCTGTTTGACCCCATCCCGACCCACG-3′) and oligo-AS (5′-TCTCGTGTTCGTCACAATGTCTACGACAAACGCAATCG-3′), respectively. The DIG-labeled oligos were detected using a chemiluminescence-based DIG detection kit (Roche), according to the manufacturer’s instructions.

### 2.5. RT-PCR

Total RNAs were extracted from the mycelia of 9-1 or 9-1C using TRIzol (Thermo-Fisher), according to the manufacturer’s instructions. One microgram of total RNA was used as template for synthesis of first-strand cDNA using 6mer random primer and reverse transcriptase SSIII (Thermo-Fisher). Two microliters of the first-strand cDNA samples were used as templates for PCR reactions using ORF1-specific primers (ORF1-F: 5′-ATGTCTACGACAAACG-3′; ORF1-R: 5′-CTAGACTTGAAAGGGATTG-3′) and Q5 DNA polymerase (NEB). PCR reactions was run in a thermocycler (Eppendorf) with 98 °C for 30 sec, 35 cycles of 98 °C for 10 s, 56 °C for 15 s, and 72 °C for 15 s, followed by an extension of 72 °C for 5 min. The resultant PCR products were separated on 1% agarose gel and visualized under UV light after ethidium bromide staining.

### 2.6. Virulence Assays

Virulence of *N. oryzae* was assayed as described previously [54], with minor modifications, conidial suspensions as inocula being replaced with mycelia-containing agar plugs. Agar plugs with diameters of 8 mm were cut from the equivalent positions of the 5-day-old 9-1 or 9-1C PDA plates and transferred to wounded leaf blades. After incubation for 2 days at 28 °C, these inoculated leaves were kept in a Petri dish at room temperature for the desired period. Virulence was assessed 14 days post inoculation (dpi) by determining the area of dark brown to black lesions using Image J [55]. The virulence experiment was performed twice independently.

### 2.7. Sequence and Phylogenetic Analyses

Searches for homologous sequences in GenBank were carried out using BLAST [56]. Conserved motifs for viral proteins were searched in the SMART and/or pfam database [57]. Phylogenetic analysis was performed according to a methodology described previously [50]. Briefly, viral protein sequences were aligned using Clustal W [58], then subjected to calculations of genetic distance using the P-distance model in MEGA 11. Finally, a phylogenetic tree was constructed using the maximum likelihood method with a bootstrap value of 1000. RNA structures were predicted by mFold [59] and redrawn by RNAdrawer 2.0 [60]. The conserved motifs of the catalytic domain of NoNRV1-ZJ RdRP were identified according to the predicted protein structure, which was modeled by Phyre2 [61]. Alignments of each motif across the chosen unsigned, non-segmented dsRNA viruses were performed using Clustal W.

## 3. Results

### 3.1. Isolation of dsRNA Segments from N. oryzae

**Figure 1:**
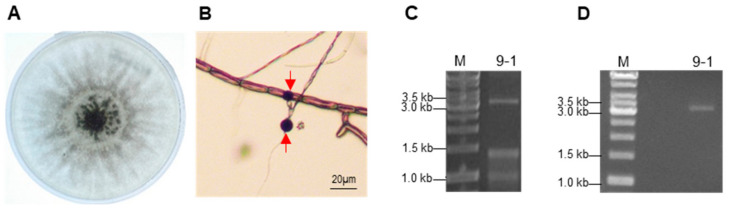
The strain 9-1 of *Nigrospore oryzae* contains a ~3 kb double-stranded (ds) RNA segment. (**A**) Colonial morphology of 9-1. (**B**) Microscopic view of hyphae with septa and conidiospores. Red arrows indicate the conidiospores. (**C**) Electrophoresis of the dsRNA sample extracted from 9-1. M indicates a DNA ladder. (**D**) Electrophoresis of the dsRNA sample after digestion by nucleases DNase I and RNase S1 simultaneously. M indicates a DNA ladder.

A filamentous fungus was isolated from a mild mosaic, surface-disinfected leaf of a chrysanthemum plant. The fungal colony initially appeared white and turned gray and black at about 5 dpi on PDA plates (Figure 1A). The filamentous hyphae were divided into cell compartments by septa (Figure 1B). On PDA plates, the fungus produced a limited number of black, spherical conidiospores with a size of about 10 µm in diameter at late growing stage (Figure 1B), which is a typical spore-bearing structure of *Nigrospora* species [62]. To determine the exact species, we amplified and sequenced the ITS sequence from the fungus (Supplementary Figure S1A). A BLAST search for the nucleotide sequence in GenBank showed that the ITS sequence has the highest identity (up to 99.24%) with the ITS sequences of *N. oryzae* isolates (Supplementary Figure S1B). Combined with the morphological characteristics, the ITS data indicate that the isolated fungus is an isolate of *N. oryzae*, designated 9-1. 

To test whether the isolate 9-1 contained mycoviral dsRNAs, dsRNAs were isolated from the isolated mycelia and separated by electrophoresis. The electrophoresis result displayed three clear bands (Figure 1C). The largest band was approximately 3 kb in size, and the other two bands were less than 1.5 kb. After digestion of the dsRNA sample with both nucleases, DNase I and RNase S1, simultaneously, only the largest band was retained (Figure 1D), indicating that 9-1 contained a ~3 kb dsRNA segment.

### 3.2. Sequence Analysis of the Mycovirus Infecting the 9-1 Isolate of N. oryzae

**Figure 2:**
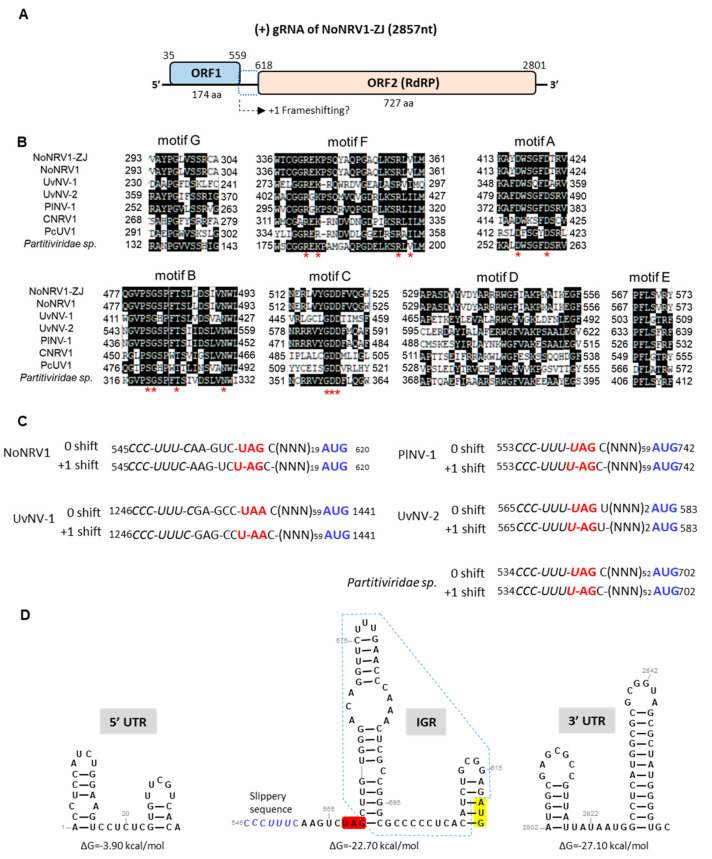
Characterization of the genomic sequence of NoNRV1-ZJ. (**A**) Genome organization. The positive strand of the NoNRV1-ZJ genome is predicted to encode two ORFs (ORF1, ORF2). The arrow with dashed lines indicates a plausible ribosomal +1 frameshifting via a slippery sequence (shown in the panel (**C**)) at the 3′ end of the ORF1. (**B**) Conserved motifs (A-G) of the catalytic domain of RdRP from unsigned, non-segmented dsRNA mycoviruses. Red asterisks indicate the conserved residues in the characterized motifs. (**C**) The proposed slippery sequences for +1 frameshifting in five non-segmented dsRNA mycoviruses. Slippery sequences are italicized. The stop codon of ORF1 is colored red. The initiation codon of ORF2 is colored blue. (NNN) indicates the non-stop codon of the intergenic sequence via ribosomal +1 frameshifting translation. (**D**) Predicted secondary structures of NoNRV1-ZJ 5′ UTR, IGR, and 3′ UTR. The IGR is surrounded by dashed lines. A possible slippery sequence for ribosomal +1 frameshifting is colored blue. The stop codon of ORF1 and the start codon of ORF2 are colored red and yellow, respectively. Free energy was calculated by mFOLD and is shown below.

To obtain the genetic information of the dsRNA molecule, the full-length cDNA was amplified and sequenced. Consensus sequence of the full-length cDNA was retrieved from two independent clones and deposited in GenBank (accession number: OK166809.1). Sequence analyses showed that the dsRNA molecule is composed of 2857 bp with a G+C composition of 57.72%. A BLAST search for the whole dsRNA sequence showed that it has a 99% identity with the previous isolate HN-21 of NoNRV1 [48], which is a monopartite dsRNA mycovirus. This indicates the *N. oryzae* 9-1 was infected with an isolate of NoNRV1, designated NoNRV1-ZJ. As reported previously [48], the positive strand of the NoNRV1-ZJ genome contains two ORFs separated by a 58nt intergenic sequence (IGR) (Figure 2A). ORF1 is located at the 5′ end of the dsRNA and encodes an approximately 19 kDa protein with no putative conserved domains and an unknown function. A BLAST search for the ORF1-encoded protein showed that it matched best with the ORF1-encoded protein of the NoNRV1 HN-21 isolate, showing a 98.9% similarity [48]. In addition, it has similarities (31.5–53.09%) with six non-segmented dsRNA mycoviruses, including *Ustilaginoidea virens nonsegmented virus 2* (UvNV-2, MH094804.1), *Ustilaginoidea virens nonsegmented virus 1* (UvNV-1, KJ605397.1), *Purpureocillium lilacinum nonsegmented virus 1* (PlNV-1, KU747155.1), *Phytophthora cactorumusti-like virus 1* (PCLV1, MW349904.1), *Conidiobolus non-segmented RNA virus 1* (CNRV-1, MT246778), and a proposed partitivirus species (MN033127). ORF2 located downstream of ORF1 encodes an approximately 83 kDa, putative RdRP (Figure 2A), with a 99.4% similarity to that of NoNRV1 HN-21 [48]. 

RdRPs have a conserved catalytic structure, which contains a set of seven motifs A-G [63]. These seven motifs were identified from the protein structure of NoNRV1-ZJ RdRP modeled by Phyre2 (Figure 2B). Sequence alignment showed that these motifs are conserved to differing extents across these seven mycoviruses (Figure 2B), whose ORF1s have a sequence similarity to that of NoNRV1. Out of these seven viruses, three viruses (UvNV-1, UvNV-2, PlNV-1) have been proposed to express their RdRP as a fusion protein together with ORF1 via a +1 ribosomal frameshift [64]. PlNV-1 and UvNV-2 have the same slippery sequence context (CCC_UUU_UAG), in which the slippery sequence has one base overlapping with the stop codon of ORF1 (Figure 2C). Sequence analysis showed that the same slippery sequence context is also present in the unknown, proposed partitivirus species isolate H1 (Figure 2C), which was determined by metatranscriptomic reconstruction in a soil sample [65]. Here, we found that NoNRV1 has a slightly different slippery sequence context, where five bases are inserted between the slippery sequence (CCC_UUU_C) and the stop codon of ORF1 (Figure 2C). This context is the same as that present in the virus UvNV-1 (Figure 2C). Thus, we speculate that NoNRV1 would use the slippery sequence to translate the ORF2 via ribosomal +1 frameshifting, producing an approximately 105 kDa fusion protein between ORF1 and ORF2. It is worth mentioning that the intergenic sequence between the two ORFs is translatable via +1 frameshifting in the five viruses mentioned above. 

The 5′ untranslated region (5′ UTR) and 3ʹ UTR of the NoNRV1-ZJ genome are 34 nt and 56 nt in length, respectively. The 5′ UTR sequence was predicted to form two short hairpins with a ∆G value of –3.90 kcal/mol (Figure 2D). The 3′ UTR sequence was folded into two hairpins with perfect base paring in their stems (Figure 2D). Such hairpin structures are common in the UTRs of other dsRNA mycoviruses [32,43,44,45,66,67] and are presumed to play an important role in viral replication and assembly [32,46,47]. The IGR sequence between ORF1 and ORF2 is 58 nt long and forms two hairpins that are separated by a 9 nt stretch (Figure 2D). The stop codon UAG of ORF1 is positioned at the 5′ base of the upstream hairpin and the slippery sequence is just 7 nt upstream of the hairpin structure. A stable hairpin structure immediately downstream of the slippery sequence is essential for induction of ribosomal-1 frameshifting. We do not know yet whether the presence of the hairpin structure(s) in the IGR is required for the slippery sequence to stimulate ribosomal +1 frameshifting. 

### 3.3. Phylogenetic Analyses of the ORF1- and ORF2-Encoded Proteins

**Figure 3:**
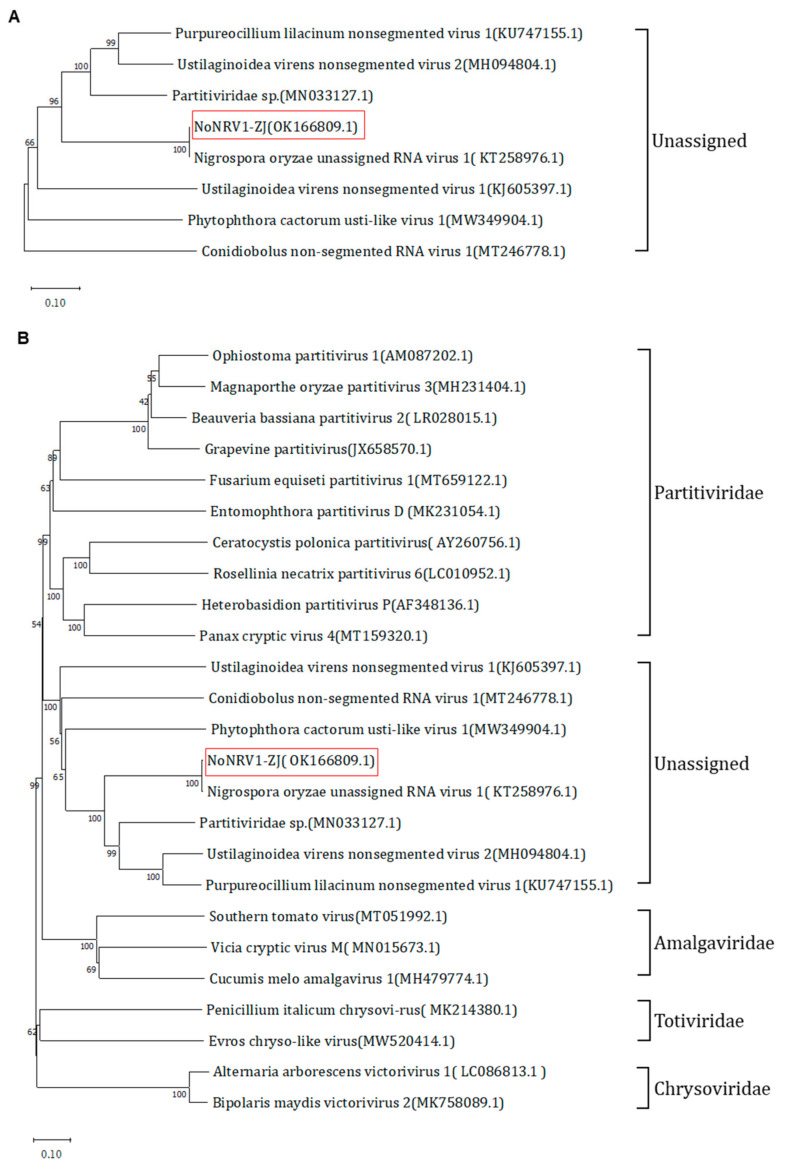
Phylogenetic analysis of NoNRV1-ZJ with other mycoviruses. (**A**) Phylogenetic tree of the ORF1-encoded protein from seven unassigned non-segmented dsRNA mycoviruses. (**B**) Phylogenetic tree of the RdRP protein encoded by five different groups of mycoviruses with dsRNA genomes. NoNRV1-ZJ (OK166809.1) identified in this work is highlighted by a red rectangle.

To determine the relationship of NoNRV1-ZJ with other mycoviruses, we constructed both phylogenetic trees based on the ORF1 or ORF2 proteins of selected viruses. It is worth noting that only seven viruses were included in the phylogenetic analysis of ORF1 because no other mycoviruses reported so far have an equivalent ORF1. These seven viruses are unassigned, non-segmented mycoviruses, with the exception of a partitivirus species isolate H1. The phylogenetic tree showed that NoNRV1 forms a clade with PlNV-1, UvNV-1, UvNV-2, and the partitivirus species (Figure 3A). Interestingly, in the phylogenetic tree of ORF2 (RdRP), NoNRV1 clustered together with six other unassigned non-segmented viruses (Figure 3B), suggesting that ORF1 evolved with less restriction than RdRP among the unassigned, non-segmented dsRNA mycoviruses. 

### 3.4. Ribavirin Treatment Removed NoNRV1-ZJ from Its Host N. oryzae

**Figure 4:**
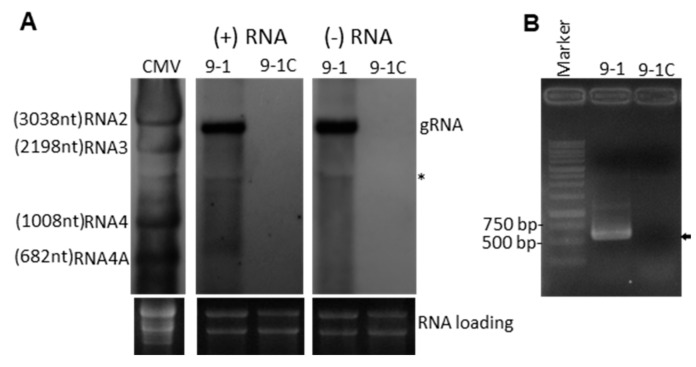
*N. oryzae* 9-1 was cured of NoNRV1-ZJ infection via Ribavirin treatment. (**A**) Detection of the positive and negative strands of NoNRV1-ZJ in untreated (9-1) and Ribavirin-treated (9-1C) mycelia by RNA gel blot. Total RNAs extracted from 9-1 and 9-1C were detected by positive strand- and negative strand-specific DNA probes. Cucumber mosaic virus (CMV) was used as an RNA ladder. The size of each CMV RNA is shown. An asterisk (*) indicates an unknown band. Ethidium bromide-stained ribosomal RNAs were used as loading controls. (**B**) Detection of NoNRV1-ZJ in 9-1 and 9-1C mycelia by RT-PCR. Total RNAs used for RNA gel blot (**A**) were used as templates for the synthesis of viral cDNAs, followed by PCR, with the primers targeting the ORF1 sequence of NoNRV1-ZJ. PCR products were separated on a 1% agarose gel. Arrow indicates the target band with expected size.

To obtain a NoNRV1-free *N. oryzae*, an attempt was made to remove the virus in the isolate 9-1 by culturing the fungus on Ribavirin-containing PDA plates with five successive passages. Afterwards, total RNAs were extracted from untreated (9-1) and Ribavirin-treated (9-1C) mycelia and used for detection of the positive and minus strands of the viral genome by RNA gel blot analysis. Here, the total RNA extracted from cucumber mosaic virus (CMV)-infected plants was used as an RNA ladder after hybridization with a CMV-specific probe. In the 9-1 sample, both positive and negative strands of NoNRV1-ZJ were detected markedly at the position slightly below CMV RNA2 (3038 nt), which is consistent with the genome size of NoNRV1-ZJ (Figure 4A). In addition, one faint unknown band (indicated by an asterisk) positioned between CMV RNA3 (2198 nt) and RNA4 (1008 nt) was detected in both hybridization reactions. Strikingly, both positive and negative strands of the viral RNA were undetectable in the drug-treated mycelia (9-1C) (Figure 4A). Detection of the viral RNA by RT-PCR showed that a DNA band with a size corresponding to that of ORF1 was detected in 9-1 but not in 9-1C (Figure 4B). Both data indicate that the drug treatment with Ribavirin successfully removed NoNRV1-ZJ from the host mycelia and provided a NoNRV1-free strain (9-1C). 

### 3.5. Infection of NoNRV1-ZJ Promoted Pigmentation of Host Cells

**Figure 5:**
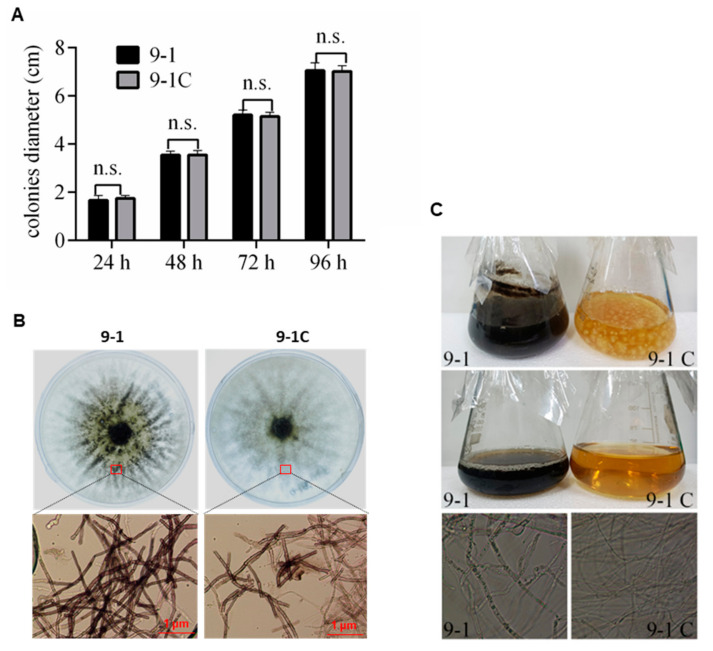
Infection with NoNRV1-ZJ promoted pigmentation of host cells. (**A**) Colonial sizes of 9-1 and 9-1C on PDA plates 4 days after inoculation. n.s. indicates no statistical difference at a certain time point. (**B**) Macroscopic and microscopic views of 9-1 and 9-1C on PDA plates at 7 days post inoculation. Upper panel: mycelium morphologies of 9-1 and 9-1C on PDA plates. Lower panel: microscopic observation of hyphae collected from both plates, as indicated by red rectangles. (**C**) Cultivation of 9-1 and 9-1C in liquid PDA media for 22 days. Top panel: liquid cultures; middle panel: filtered liquid from the liquid cultures; bottom panel: microscopic observation of the hyphae recovered from the liquid cultures.

To investigate the potential effects of NoNRV1 infection on host growth and morphology, we compared the growth of 9-1 and 9-1C on PDA plates. Their colonial diameters were not statistically different at 24, 48, 72, or 96 h post inoculation (Figure 5A), indicating no discernable effects of virus infection on the growth rate of its host. Interestingly, an obvious difference in colony morphology appeared at 7 dpi. The 9-1 colonies showed a broad yellowish zone with scattered dark patches in the area surrounding the central inoculum, while the 9-1C colonies just had a much smaller yellowish area with limited dark patches (Figure 5B, upper panel). Microscopic observation of hyphae collected at the equivalent position in both plates showed that the 9-1 hyphae were much darker than those of 9-1C, and no spores were observed in either culture (Figure 5B, lower panel), suggesting that NoNRV1 infection promoted host hyphal pigmentation. Such a difference was also observed in 22-day-old liquid cultures (Figure 5C, top panel). The liquid culture of 9-1 was dark but that of 9-1C was yellow, like the original color of the PDA media. After vacuum filtration, the filtered liquid of 9-1 was dark (Figure 5C, middle panel), as observed in its liquid culture, suggesting that the black substance produced by 9-1 was soluble in liquid media. Microscopic observation of the hyphae recovered from the liquid culture showed that both hyphae of 9-1 and 9-1C were quite transparent, while the 9-1 cells contained many vesicle structures (Figure 5C, bottom panel). Taken together, our results demonstrate that NoNRV1 infection has no marked impact on host growth rate but promotes host cell pigmentation. The cell pigmentation might be caused by a hydrophilic secondary metabolite, the biosynthesis regulated by NoNRV1 infection. 

### 3.6. NoRV1-ZJ Reduced N.oryzae Heat Tolerance

**Figure 6:**
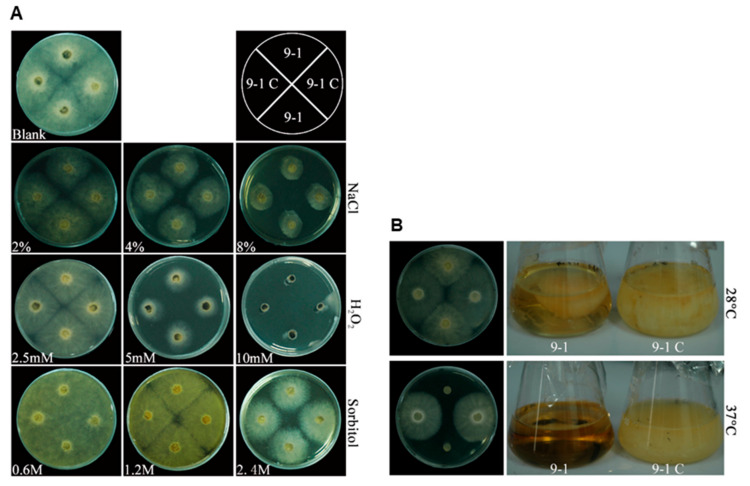
Effect of NoNRV1-ZJ infection on the growth of fungal hosts under different stress conditions. (**A**) The growth of 9-1 and 9-1C in the PDA plates containing different concentrations of NaCl, sorbital, or H_2_O_2_. The schematic diagram shown at the top right indicates the inoculating pattern of both cultures in plates. (**B**) The growth of 9-1 and 9-1C in the PDA plate or the liquid media under 28 °C or 37 °C. All the plates were photographed at 4 dpi, and the liquid cultures were photographed at 7 dpi.

Previous work reported that the pigment melanin protects fungi from hostile environmental stresses, such as high temperatures [68,69], heavy metals [70,71], and high radiation levels [22]. Thus, we wondered whether the increased pigmentation of *N. oryzae* caused by infection with NoNRV1 confers any benefits on the fungus against various abiotic stresses. Here, we tested the growth of 9-1 and 9-1C on the PDA plates containing different concentrations of osmotic (NaCl, sorbitol) or oxidative (H_2_O_2_) chemicals. Each chemical at a high concentration showed obvious inhibition of the growth of both fungal cultures, and 9-1 had no growth advantages over 9-1C on the chemical-containing plates (Figure 6A), suggesting that pigmentation caused by the virus infection does not improve host tolerance to these abiotic stresses.

Then, we tested the effects of incubating temperatures on both 9-1 and 9-1C. Both cultures grew well and almost the same on PDA plates at 28 °C (Figure 6B, upper panel). However, their growth differed completely at 37 °C (Figure 6B, lower panel). The 9-1 isolate could not grow, while 9-1C grew as well as it did at 28 °C. Experiments with liquid media further demonstrate that 9-1C, but not 9-1, could grow at 37 °C (Figure 6B, lower panel). In the liquid cultures, it seems that 9-1C produced more biomass than 9-1 at 28 °C. However, we did not see much difference in biomass between them in a biological repeat (Supplementary Figure S2). Thus, it can be concluded that 9-1C does not produce biomass more than 9-1. All these data demonstrate that *N. oryzae* itself is thermotolerant at 37 °C, while NoNRV1 infection compromises its tolerant capability in such harsh conditions.

### 3.7. NoRV1-ZJ Caused Hypovirulence to N. oryzae

**Figure 7:**
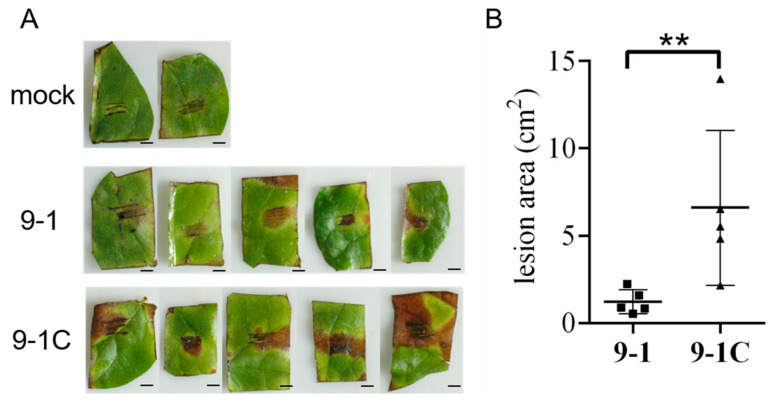
NoNRV1-ZJ caused hypovirulence to *N.oryzae* in cotton leaves. (**A**) Local lesion diseases of cotton leaves with inoculation of 9-1 or 9-1C. Mock was the wounded cotton leaves treated with water. The disease symptoms were photographed at 12 dpi. Bar represents 1 cm. (**B**) Statistic analyses of local lesion areas between 9-1 and 9-1C. ** indicates a *p*-value < 0.01, calculated by Student’s *t*-test.

*N. oryzae* has been reported to be a cotton-infecting pathogen, causing disease symptoms [36,72]. To investigate the plausible effects of NoNRV1 infection on the pathogenicity of *N. oryzae*, we tested infection of 9-1 and 9-1C on wounded cotton leaves. As reported, both 9-1 and 9-1C caused brown necrotic lesions on all the tested leaves at 12 dpi (Figure 7A). Interestingly, the lesions caused by 9-1C were significantly larger than those caused by 9-1 (Figure 7B), indicating that the infection with NoRV1-ZJ led to hypovirulence of the fungal host in the cotton leaves.

## 4. Discussion

In this work, we determined the infection of NoNRV1 in the fungus *N. oryzae* and successfully removed the virus from the isolated fungus by drug treatment, which provided us with an ideal tool to investigate the impact of NoNRV1 infection on the fungal host. By comparing the original and virus-cured strains, our results demonstrated that NoNRV1 infection promotes host cell pigmentation, makes its host lose viability at high temperatures (37 °C), and alleviates host virulence in cotton leaves. Our work provides phenotypic evidence that uncovers the biological impacts of NoNRV1 infection on fungal hosts.

The NoNRV1 genome encodes two separated ORFs. It is a mystery how the 3′ proximal ORF2 is translated. Non-canonical translational strategies, including translation stop/restart, and ribosomal −1 or +1 frameshifting have been identified as the molecular mechanisms used by some mycoviruses to translate 3′ proximal ORFs [73,74,75,76,77]. NoNRV1 could not use the stop/restart mechanism since it lacks a typical stop/restart motif such as *AUG*A or UA*A**UG* (underlined: ORF1 stop codon; italicized: ORF2 start codon) at the ORF1/ORF2 junction that has been illustrated well in the *Helminthosporium victoriae virus 190S* [75,76]. It has been reported that Totiviruses have a well characterized slippery sequence for ribosomal-1 frameshifting that causes a fusion event between gag and pol proteins [66,67,74]. Interestingly, NoNRV1 has a similar slippery sequence (CCC_UUU_C) to other unassigned mycoviruses (PlNV-1, UvNV-1, UvNV-2), just 5 nt ahead of the ORF1 stop codon (Figure 2C). We also found that such a slippery sequence is present in a proposed partitivirus species isolate H1 (Figure 2C). Moreover, influenza A virus was reported to use the slippery sequence for +1 frameshifting to express the PA-X gene [78]. Thus, we speculate that +1 frameshifting is the molecular tactic used by these unassigned, non-segmented mycoviruses (including NoNRV1) for production of their RdRP protein. However, we did not find a +1 slippery sequence in PCUV-1 or CRNV-1, which are genetically close to these unassigned mycoviruses (Figure 3B). The absence of such a sequence suggests that both unassigned, non-segmented viruses PCUV-1 and CRNV-1 use a different molecular mechanism to produce their RdRP. 

Here, we reported the interesting phenomenon that NoNRV1 infection promoted pigmentation in host hyphae on solid media (Figure 5B). A similar impact has been reported previously in *C. protuberata* with infection by CThTV [32]. CThTV infection markedly increases melanin content in host cells, leading to darkened mycelia. Generally, the brown-black pigments produced by microorganisms are melanin or melanin-like pigments [79,80]. However, two lines of our experimental data make us uncertain that the enhanced pigmentation of *N. oryzae* mycelia in the presence of NoNRV1 is the outcome of increased melanin. Firstly, melanin is important for fungi to protect themselves from adverse stresses [81], while NoNRV1 infection accompanied with increased pigmentation does not improve its host’s abilities to withstand osmotic or oxidative stresses (Figure 6A). Secondly, as a class of polymerized and hydrophobic pigments with high molecular weight, melanin is completely insoluble in almost all solvents [82]. However, we observed that the black substance produced by the strain 9-1 in liquid culture is hydrophilic (Figure 6B), which is inconsistent with the physical property of melanin. Another speculation concerning the promoted pigmentation is that NoNRV1 infection alters host metabolism to produce a specific secondary metabolite that causes host cell pigmentation. Thus, it would be interesting to determine the black substance in the liquid culture as this will shed light on the underlying basis of how NoNRV1 infection promotes pigmentation in host cells. 

Hypovirulence is one of the most fascinating phenomena occurring through interactions between mycoviruses and fungal hosts. Recently, two mycoviruses (*Pestalotiopsis theae chrysovirus-1*, PtCV1; *Sclerotinia sclerotiorum hypovirulenceassociated DNA virus 1*, SsHADV-1) were reported to be hypovirulence-inducing agents which convert their pathogenic fungal hosts into non-pathogenic, even beneficial endophytes [83,84,85]. Hypovirulence may be associated with alterations in the morphology, pigmentation, sporulation, and growth rate of fungal hosts. It can be explained simply as the direct outcome of resource appropriation between mycoviruses and hosts during viral replication [86]. Zhang et al. [85] reported that SsHADV-1 compromises host virulence by repressing the expression of key pathogenesis factor genes in host cells. Here, we have reported a similar phenotype—that NoNRV1 reduced *N. oryzae* pathogenicity in cotton leaves (Figure 7)—but the underlying mechanism remains to be investigated. 

The relationship between mycovirus and fungal heat-resistance is complicated. Previously, CThTV has been reported to enhance fungal host heat-tolerance [20], which could be associated with fungal melanin, osmoprotectants, and heat shock proteins [21]. Recently, Olivé et al. [87] reported an inhibitory impact of *Colletorichum higginsianum non-segmented virus* (ChNRV1) on its fungal host at a high temperature (32 °C). This is similar to our finding that NoNRV1 infection made its host completely lost thermotolerance at 37 °C (Figure 6B). Strikingly, the NoNRV1-free *N. oryzae* isolate (9-1C) grew as well at 37 °C as did at 28 °C, suggesting that *N. oryzae* itself is extremely thermotolerant. Exposure to high temperature is a useful way to cure mycoviruses in their hosts [88,89]. Thus, we hypothesize that NoNRV1 impairs host heat-resistance to prevent the generation of a virus-free strain under high temperatures, which would be beneficial for maintaining symbiosis between the virus and its hosts.

## Supplementary Materials

The following supporting information can be downloaded at: https://www.mdpi.com/article/10.3390/v14040795/s1, Figure S1: Molecular identification of the isolated fungus 9-1. The DNA sequence of internal transcribed spacer 1 of 9-1 (A), and top 10 homologous sequences in NCBI GenBank via BLAST search (B). Figure S2: Liquid cultures of 9-1 and 9-1C in PDA media at 7 dpi.

## References

[1] Son M., Yu J., Kim K.-H. (2015). Five questions about mycoviruses. PLoS Pathog..

[2] Ai Y.P., Zhong J., Chen C.Y., Zhu H.J., Gao B.D. (2016). A novel single-stranded RNA virus isolated from the rice-pathogenic fungus *Magnaporthe oryzae* with similarity to members of the family Tombusviridae. Arch. Virol..

[3] Canizares M.C., Lopez-Escudero F.J., Perez-Artes E., Garcia-Pedrajas M.D. (2018). Characterization of a novel single-stranded RNA mycovirus related to invertebrate viruses from the plant pathogen *Verticillium dahliae*. Arch. Virol..

[4] Khan H.A., Sato Y., Kondo H., Jamal A., Bhatti M.F., Suzuki N. (2021). A second capsidless hadakavirus strain with 10 positive-sense single-stranded RNA genomic segments from *Fusarium nygamai*. Arch. Virol..

[5] Liu H., Wang H., Lu X., Xiao C., Peng B., Zhou Q. (2021). Molecular characterization of a novel single-stranded RNA virus, ChRV1, isolated from the plant-pathogenic fungus *Colletotrichum higginsianum*. Arch. Virol..

[6] Liu R., Cheng J., Fu Y., Jiang D., Xie J. (2015). Molecular Characterization of a Novel Positive-Sense, Single-Stranded RNA Mycovirus Infecting the Plant Pathogenic Fungus *Sclerotinia sclerotiorum*. Viruses.

[7] Sato Y., Shamsi W., Jamal A., Bhatti M.F., Kondo H., Suzuki N. (2020). Hadaka Virus 1: A Capsidless Eleven-Segmented Positive-Sense Single-Stranded RNA Virus from a Phytopathogenic Fungus, *Fusarium oxysporum*. mBio.

[8] Zhang R., Liu S., Chiba S., Kondo H., Kanematsu S., Suzuki N. (2014). A novel single-stranded RNA virus isolated from a phytopathogenic filamentous fungus, *Rosellinia necatrix*, with similarity to hypo-like viruses. Front. Microbiol..

[9] Zhong J., Shang H.H., Zhu C.X., Zhu J.Z., Zhu H.J., Hu Y., Gao B.D. (2016). Characterization of a novel single-stranded RNA virus, closely related to fusariviruses, infecting the plant pathogenic fungus *Alternaria brassicicola*. Virus Res..

[10] Yu X., Li B., Fu Y., Jiang D., Ghabrial S.A., Li G., Peng Y., Xie J., Cheng J., Huang J. (2010). A geminivirus-related DNA mycovirus that confers hypovirulence to a plant pathogenic fungus. Proc. Natl. Acad. Sci. USA.

[11] McCabe P.M., Pfeiffer P., Van Alfen N.K. (1999). The influence of dsRNA viruses on the biology of plant pathogenic fungi. Trends Microbiol..

[12] Hillman B.I., Annisa A., Suzuki N. (2018). Viruses of Plant-Interacting Fungi. Adv. Virus Res..

[13] Park M., Cho Y.-J., Kim D., Yang C.-S., Lee S.M., Dawson T.L., Nakamizo S., Kabashima K., Lee Y.W., Jung W.H. (2020). A Novel Virus Alters Gene Expression and Vacuolar Morphology in Malassezia Cells and Induces a TLR3-Mediated Inflammatory Immune Response. mBio.

[14] McBride R.C., Boucher N., Park D.S., Turner P.E., Townsend J.P. (2013). Yeast response to LA virus indicates coadapted global gene expression during mycoviral infection. FEMS Yeast Res..

[15] Lukša J., Ravoitytė B., Konovalovas A., Aitmanaitė L., Butenko A., Yurchenko V., Serva S., Servienė E. (2017). Different Metabolic Pathways Are Involved in Response of *Saccharomyces cerevisiae* to L-A and M Viruses. Toxins.

[16] Ghabrial S.A., Suzuki N. (2009). Viruses of plant pathogenic fungi. Annu. Rev. Phytopathol..

[17] Nuss D.L. (2005). Hypovirulence: Mycoviruses at the fungal–plant interface. Nat. Rev. Microbiol..

[18] Coelho V., Nunes L., Gouveia E. (2021). Short and long term efficacy and prevalence of *Cryphonectria parasitica* hypovirulent strains released as biocontrol agents of chestnut blight. Eur. J. Plant Pathol..

[19] Chen B., Nuss D.L. (1999). Infectious cDNA Clone of Hypovirus CHV1-Euro7: A Comparative Virology Approach to Investigate Virus-Mediated Hypovirulence of the Chestnut Blight Fungus *Cryphonectria parasitica*. J. Virol..

[20] Márquez L.M., Redman R.S., Rodriguez R.J., Roossinck M.J. (2007). A virus in a fungus in a plant: Three-way symbiosis required for thermal tolerance. Science.

[21] Morsy M.R., Oswald J., He J., Tang Y., Roossinck M.J. (2010). Teasing apart a three-way symbiosis: Transcriptome analyses of *Curvularia protuberata* in response to viral infection and heat stress. Biochem. Bioph. Res. Commun..

[22] Dadachova E., Casadevall A. (2008). Ionizing radiation: How fungi cope, adapt, and exploit with the help of melanin. Curr. Opin. Microbiol..

[23] Hottiger T., Boller T., Wiemken A. (1987). Rapid changes of heat and desiccation tolerance correlated with changes of trehalose content in *Saccharomyces cerevisiae* cells subjected to temperature shifts. FEBS Lett..

[24] Cordero R.J., Casadevall A. (2017). Functions of fungal melanin beyond virulence. Fungal Biol. Rev..

[25] Xie J., Xiao X., Fu Y., Liu H., Cheng J., Ghabrial S.A., Li G., Jiang D. (2011). A novel mycovirus closely related to hypoviruses that infects the plant pathogenic fungus *Sclerotinia sclerotiorum*. Virology.

[26] Filippou C., Diss R.M., Daudu J.O., Coutts R.H.A., Kotta-Loizou I. (2021). The Polymycovirus-Mediated Growth Enhancement of the Entomopathogenic Fungus *Beauveria bassiana* Is Dependent on Carbon and Nitrogen Metabolism. Front. Microbiol..

[27] Özkan S., Coutts R.H. (2015). Aspergillus fumigatus mycovirus causes mild hypervirulent effect on pathogenicity when tested on *Galleria mellonella*. Fungal Genet. Biol..

[28] Choi G.H., Nuss D.L. (1992). A viral gene confers hypovirulence-associated traits to the chestnut blight fungus. EMBO J..

[29] Eisenman H.C., Casadevall A. (2012). Synthesis and assembly of fungal melanin. Appl. Microbiol. Biotechnol..

[30] Eisenman H.C., Nosanchuk J.D., Webber J.B.W., Emerson R.J., Camesano T.A., Casadevall A. (2005). Microstructure of cell wall-associated melanin in the human pathogenic fungus *Cryptococcus neoformans*. Biochemistry.

[31] Kogej T., Stein M., Volkmann M., Gorbushina A.A., Galinski E.A., Gunde-Cimerman N. (2007). Osmotic adaptation of the halophilic fungus *Hortaea werneckii*: Role of osmolytes and melanization. Microbiology.

[32] Liu C., Cleckler B., Morsy M. (2018). Development of an Expression Vector to Overexpress or Downregulate Genes in *Curvularia protuberata*. J. Fungi.

[33] Standen J. (1945). *Nigrospora oryzae* (B. and Br.) Fetch on Maize. Phytopathology.

[34] Liu L., Zhao K., Zhao Y., Zhang Y., Fu Q., Shiwen H. (2021). *Nigrospora oryzae* Causing Panicle Branch Rot Disease on *Oryza sativa* (rice). Plant Dis..

[35] Fakhrunnisa M.H., Ghaffar A. (2006). Seed-borne mycoflora of wheat, sorghum and barley. Pak. J. Bot..

[36] Zhang L.X., Li S.S., Tan G.J., Shen J.T., He T. (2012). First Report of *Nigrospora oryzae* Causing Leaf Spot of Cotton in China. Plant Dis..

[37] Chen X., Wang N., Yang M.-F., Li H.-X. (2019). First Report of Nigrospora Leaf Spot Caused by *Nigrospora oryzae* on Watermelon in China. Plant Dis..

[38] Wu J., Zhang C., Mao P., Qian Y., Wang H. (2014). First report of leaf spot caused by *Nigrospora oryzae* on *Dendrobium candidum* in China. Plant Dis..

[39] Zheng L., Shi F., Kelly D., Hsiang T. (2012). First report of leaf spot of Kentucky bluegrass (*Poa pratensis*) caused by *Nigrospora oryzae* in Ontario. Plant Dis..

[40] Li L., Pan H., Chen M., Zhang S., Zhong C. (2018). First report of *Nigrospora oryzae* causing brown/black spot disease of Kiwifruit in China. Plant Dis..

[41] Han S., Yu S., Zhu T., Li S., Qiao T., Liu Y., Lin T., Yang C. (2021). *Nigrospora oryzae* Causing Black Leaf Spot Disease of *Hibiscus mutabilis* in China. Plant Dis..

[42] Sharma P., Meena P.D., Chauhan J.S. (2013). First Report of *Nigrospora oryzae* (Berk & Broome) Petch Causing Stem Blight on *Brassica juncea* in India. J. Phytopathol..

[43] Wang Y., Jingai Y., Li Z., Huo J., Zhou S., Liu W., Wu H. (2021). Genome Sequence Resource for *Nigrospora oryzae*, an important pathogenic fungus threatening crop production. Mol. Plant Microbe Interact..

[44] Zhong J., Zhou Q., Hu Y., Zhu H.J., Da Gao B. (2016). Molecular identification of a novel victorivirus from the phytopathogenic fungus *Nigrospora oryzae*. Virus Genes.

[45] Liu H., Liu R., Li C.X., Wang H., Zhu H.J., Gao B.D., Zhou Q., Zhong J. (2019). A victorivirus and two novel mitoviruses Co-infected the plant pathogen *Nigrospora oryzae*. Viruses.

[46] Zhong J., Zhao S.Q., Li G.F., Pang X.D., Deng X.J., Zhu H.J., Da Gao B., Zhou Q. (2016). A novel fusarivirus isolated from the phytopathogenic fungus *Nigrospora oryzae*. Virus Genes.

[47] Yu J.X., Zhu J.Z., Wang Y., Zhang C.J., Zhong J., Zhu H.J., Da Gao B., Zhou Q. (2018). Molecular characterization of a putative gammapartitivirus in the phytopathogenic fungus *Nigrospora oryzae*. Arch. Virol..

[48] Zhou Q., Zhong J., Hu Y., Da Gao B. (2016). A novel nonsegmented double-stranded RNA mycovirus identified in the phytopathogenic fungus *Nigrospora oryzae* shows similarity to partitivirus-like viruses. Arch. Virol..

[49] Chen H., Qi Y., He X., Xu L., Zhang W., Lv X., Zhang H., Yang D., Zhu Y., Liang Z. (2021). Endophytic fungus Mucor circinelloides DF20 promote tanshinone biosynthesis and accumulation in *Salvia miltiorrhiza* root. Plant Sci..

[50] Li L., Tian Q., Du Z., Duns G.J., Chen J. (2009). A novel double-stranded RNA virus detected in Primula malacoides is a plant-isolated partitivirus closely related to partitivirus infecting fungal species. Arch. Virol..

[51] Parker W.B. (2005). Metabolism and antiviral activity of ribavirin. Virus Res..

[52] Herrero N., Zabalgogeazcoa I. (2011). Mycoviruses infecting the endophytic and entomopathogenic fungus *Tolypocladium cylindrosporum*. Virus Res..

[53] Du Z., Chen A., Chen W., Westwood J.H., Baulcombe D.C., Carr J.P. (2014). Using a viral vector to reveal the role of microRNA159 in disease symptom induction by a severe strain of *Cucumber mosaic* virus. Plant Physiol..

[54] Jia Y., Valent B., Lee F. (2003). Determination of host responses to *Magnaporthe grisea* on detached rice leaves using a spot inoculation method. Plant Dis..

[55] Rueden C.T., Schindelin J., Hiner M.C., DeZonia B.E., Walter A.E., Arena E.T., Eliceiri K.W. (2017). ImageJ2: ImageJ for the next generation of scientific image data. BMC Bioinform..

[56] Altschul S.F., Gish W., Miller W., Myers E.W., Lipman D.J. (1990). Basic local alignment search tool. J. Mol. Biol..

[57] Letunic I., Bork P. (2017). 20 years of the SMART protein domain annotation resource. Nucleic Acids Res..

[58] Thompson J.D., Higgins D.G., Gibson T.J. (1994). CLUSTAL W: Improving the sensitivity of progressive multiple sequence alignment through sequence weighting, position-specific gap penalties and weight matrix choice. Nucleic Acids Res..

[59] Zuker M. (2003). Mfold web server for nucleic acid folding and hybridization prediction. Nucleic Acids Res..

[60] Johnson P.Z., Kasprzak W.K., Shapiro B.A., Simon A.E. (2019). RNA2Drawer: Geometrically strict drawing of nucleic acid structures with graphical structure editing and highlighting of complementary subsequences. RNA Biol..

[61] Kelley L.A., Mezulis S., Yates C.M., Wass M.N., Sternberg M.J. (2015). The Phyre2 web portal for protein modeling, prediction and analysis. Nat. Protoc..

[62] Wang M., Liu F., Crous P., Cai L. (2017). Phylogenetic reassessment of Nigrospora: Ubiquitous endophytes, plant and human pathogens. Persoonia.

[63] Venkataraman S., Prasad B.V., Selvarajan R. (2018). RNA dependent RNA polymerases: Insights from structure, function and evolution. Viruses.

[64] Herrero N. (2016). A novel monopartite dsRNA virus isolated from the entomopathogenic and nematophagous fungus *Purpureocillium lilacinum*. Arch. Virol..

[65] Starr E.P., Nuccio E.E., Pett-Ridge J., Banfield J.F., Firestone M.K. (2019). Metatranscriptomic reconstruction reveals RNA viruses with the potential to shape carbon cycling in soil. Proc. Natl. Acad. Sci. USA.

[66] Baeza M., Bravo N., Sanhueza M., Flores O., Villarreal P., Cifuentes V. (2012). Molecular characterization of totiviruses in *Xanthophyllomyces dendrorhous*. Virol. J..

[67] Lee M.D., Creagh J.W., Fredericks L.R., Crabtree A.M., Patel J.S., Rowley P.A. (2022). The Characterization of a Novel Virus Discovered in the Yeast *Pichia membranifaciens*. Viruses.

[68] Ruan L., Yu Z., Fang B., He W., Wang Y., Shen P. (2004). Melanin pigment formation and increased UV resistance in *Bacillus thuringiensis* following high temperature induction. Syst. Appl. Microbiol..

[69] Wang Y., Hu X., Fang Y., Anchieta A., Goldman P.H., Hernandez G., Klosterman S.J. (2018). Transcription factor VdCmr1 is required for pigment production, protection from UV irradiation, and regulates expression of melanin biosynthetic genes in *Verticillium dahliae*. Microbiology.

[70] Chan W.K., Wildeboer D., Garelick H., Purchase D. (2016). Mycoremediation of heavy metal/metalloid-contaminated soil: Current understanding and future prospects. Fungal Applications in Sustainable Environmental Biotechnology.

[71] Łopusiewicz Ł. (2018). The isolation, purification and analysis of the melanin pigment extracted from *Armillaria mellea* rhizomorphs. World Sci. News.

[72] Palmateer A., McLean K., Van Santen E., Morgan-Jones G. (2003). Occurrence of Nigrospora lint rot caused by *Nigrospora oryzae* on cotton in Alabama. Plant Dis..

[73] Jamal A., Sato Y., Shahi S., Shamsi W., Kondo H., Suzuki N. (2019). Novel victorivirus from a Pakistani isolate of *Alternaria alternata* lacking a typical translational stop/restart sequence signature. Viruses.

[74] Dinman J.D., Icho T., Wickner R.B. (1991). A-1 ribosomal frameshift in a double-stranded RNA virus of yeast forms a gag-pol fusion protein. Proc. Natl. Acad. Sci. USA.

[75] Li H., Havens W.M., Nibert M.L., Ghabrial S.A. (2011). RNA sequence determinants of a coupled termination-reinitiation strategy for downstream open reading frame translation in *Helminthosporium victoriae* virus 190S and other victoriviruses (Family Totiviridae). J. Virol..

[76] Li H., Havens W.M., Nibert M.L., Ghabrial S.A. (2015). An RNA cassette from *Helminthosporium victoriae* virus 190S necessary and sufficient for stop/restart translation. Virology.

[77] Nibert M.L., Pyle J.D., Firth A.E. (2016). A+ 1 ribosomal frameshifting motif prevalent among plant amalgaviruses. Virology.

[78] Firth A., Jagger B., Wise H., Nelson C., Parsawar K., Wills N., Napthine S., Taubenberger J., Digard P., Atkins J. (2012). Ribosomal frameshifting used in influenza A virus expression occurs within the sequence UCC_UUU_CGU and is in the+ 1 direction. Open Biol..

[79] Durán N., Teixeira M.F., De Conti R., Esposito E. (2002). Ecological-friendly pigments from fungi. Crit. Rev. Food Sci..

[80] Vasanthabharathi V., Jayalakshmi S. (2020). Review on melanin from marine actinomycetes. J. Basic Appl. Sci..

[81] Gessler N.N., Egorova A.S., Belozerskaya T.A. (2014). Melanin pigments of fungi under extreme environmental conditions (Review). Appl. Biochem. Microbiol..

[82] Nosanchuk J.D., Stark R.E., Casadevall A. (2015). Fungal Melanin: What do We Know About Structure?. Front. Microbiol..

[83] Yu X., Li B., Fu Y., Xie J., Cheng J., Ghabrial S.A., Li G., Yi X., Jiang D. (2013). Extracellular transmission of a DNA mycovirus and its use as a natural fungicide. Proc. Natl. Acad. Sci. USA.

[84] Zhou L., Li X., Kotta-Loizou I., Dong K., Li S., Ni D., Hong N., Wang G., Xu W. (2021). A mycovirus modulates the endophytic and pathogenic traits of a plant associated fungus. ISME J..

[85] Zhang H., Xie J., Fu Y., Cheng J., Qu Z., Zhao Z., Cheng S., Chen T., Li B., Wang Q. (2020). A 2-kb mycovirus converts a pathogenic fungus into a beneficial endophyte for brassica protection and yield enhancement. Mol. Plant.

[86] Kotta-Loizou I. (2021). Mycoviruses and their role in fungal pathogenesis. Curr. Opin. Microbiol..

[87] Olivé M., Campo S. (2021). The dsRNA mycovirus ChNRV1 causes mild hypervirulence in the fungal phytopathogen *Colletotrichum higginsianum*. Arch. Microbiol..

[88] Applen Clancey S., Ruchti F., LeibundGut-Landmann S., Heitman J., Ianiri G. (2020). A Novel mycovirus evokes transcriptional rewiring in the fungus malassezia and stimulates beta interferon production in macrophages. mBio.

[89] Sasai S., Tamura K., Tojo M., Herrero M.-L., Hoshino T., Ohki S.T., Mochizuki T. (2018). A novel non-segmented double-stranded RNA virus from an Arctic isolate of *Pythium polare*. Virology.

