# Supplementary material for: Infection of Nigrospora nonsegmented RNA Virus 1 Has Important Biological Impacts on a Fungal Host
Source: Viruses. 2022 Apr 12;14(4):795. doi: 10.3390/v14040795 (PMC9029208; doi:10.3390/v14040795)
Supplement: Supplementary file 1 [file viruses-14-00795-s001.zip › viruses-1600550-supplementary.pptx]

## Slide 1
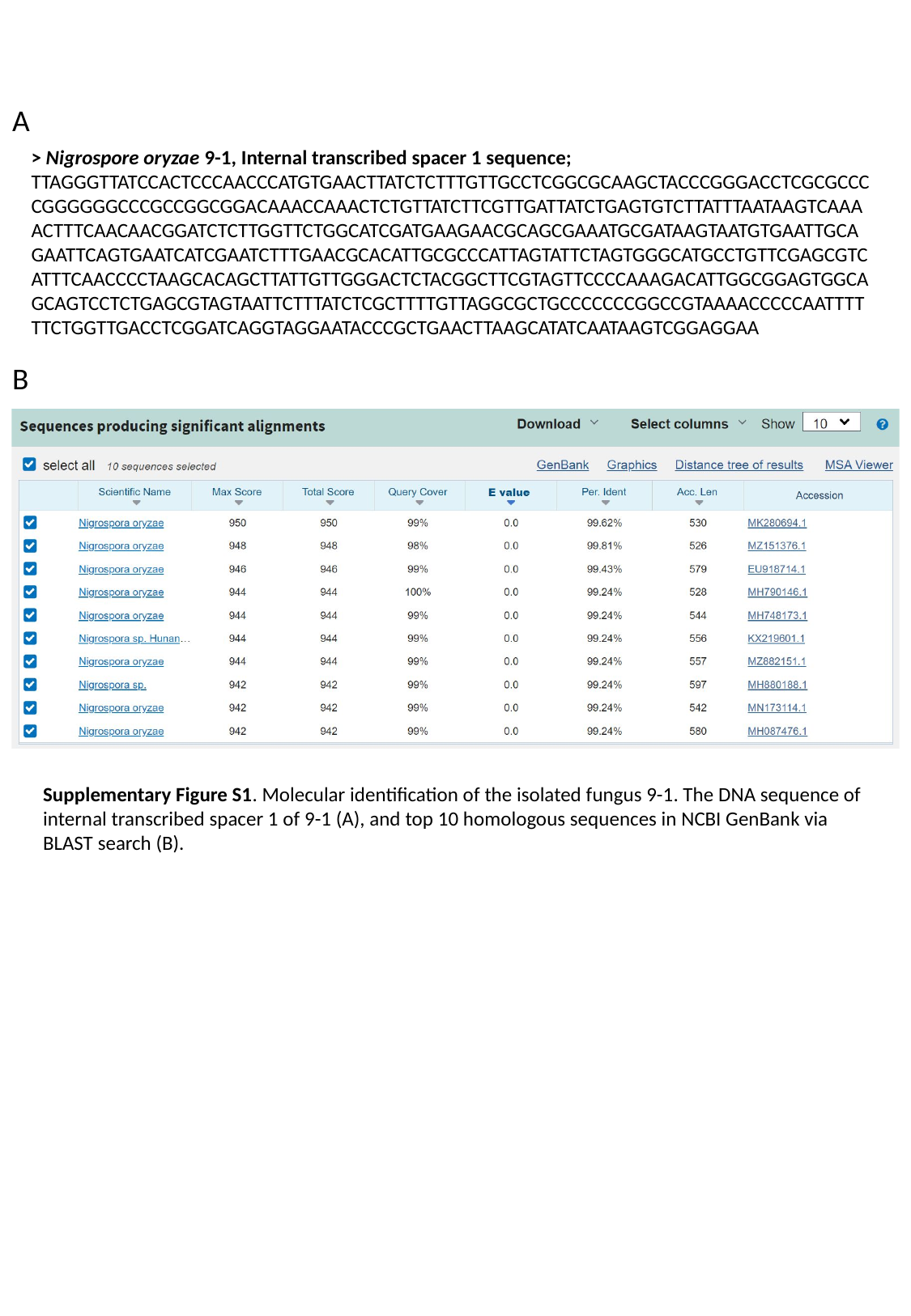

A
> Nigrospore oryzae 9-1, Internal transcribed spacer 1 sequence;
TTAGGGTTATCCACTCCCAACCCATGTGAACTTATCTCTTTGTTGCCTCGGCGCAAGCTACCCGGGACCTCGCGCCCCGGGGGGCCCGCCGGCGGACAAACCAAACTCTGTTATCTTCGTTGATTATCTGAGTGTCTTATTTAATAAGTCAAAACTTTCAACAACGGATCTCTTGGTTCTGGCATCGATGAAGAACGCAGCGAAATGCGATAAGTAATGTGAATTGCAGAATTCAGTGAATCATCGAATCTTTGAACGCACATTGCGCCCATTAGTATTCTAGTGGGCATGCCTGTTCGAGCGTCATTTCAACCCCTAAGCACAGCTTATTGTTGGGACTCTACGGCTTCGTAGTTCCCCAAAGACATTGGCGGAGTGGCAGCAGTCCTCTGAGCGTAGTAATTCTTTATCTCGCTTTTGTTAGGCGCTGCCCCCCCGGCCGTAAAACCCCCAATTTTTTCTGGTTGACCTCGGATCAGGTAGGAATACCCGCTGAACTTAAGCATATCAATAAGTCGGAGGAA
B
Supplementary Figure S1. Molecular identification of the isolated fungus 9-1. The DNA sequence of internal transcribed spacer 1 of 9-1 (A), and top 10 homologous sequences in NCBI GenBank via BLAST search (B).

## Slide 2
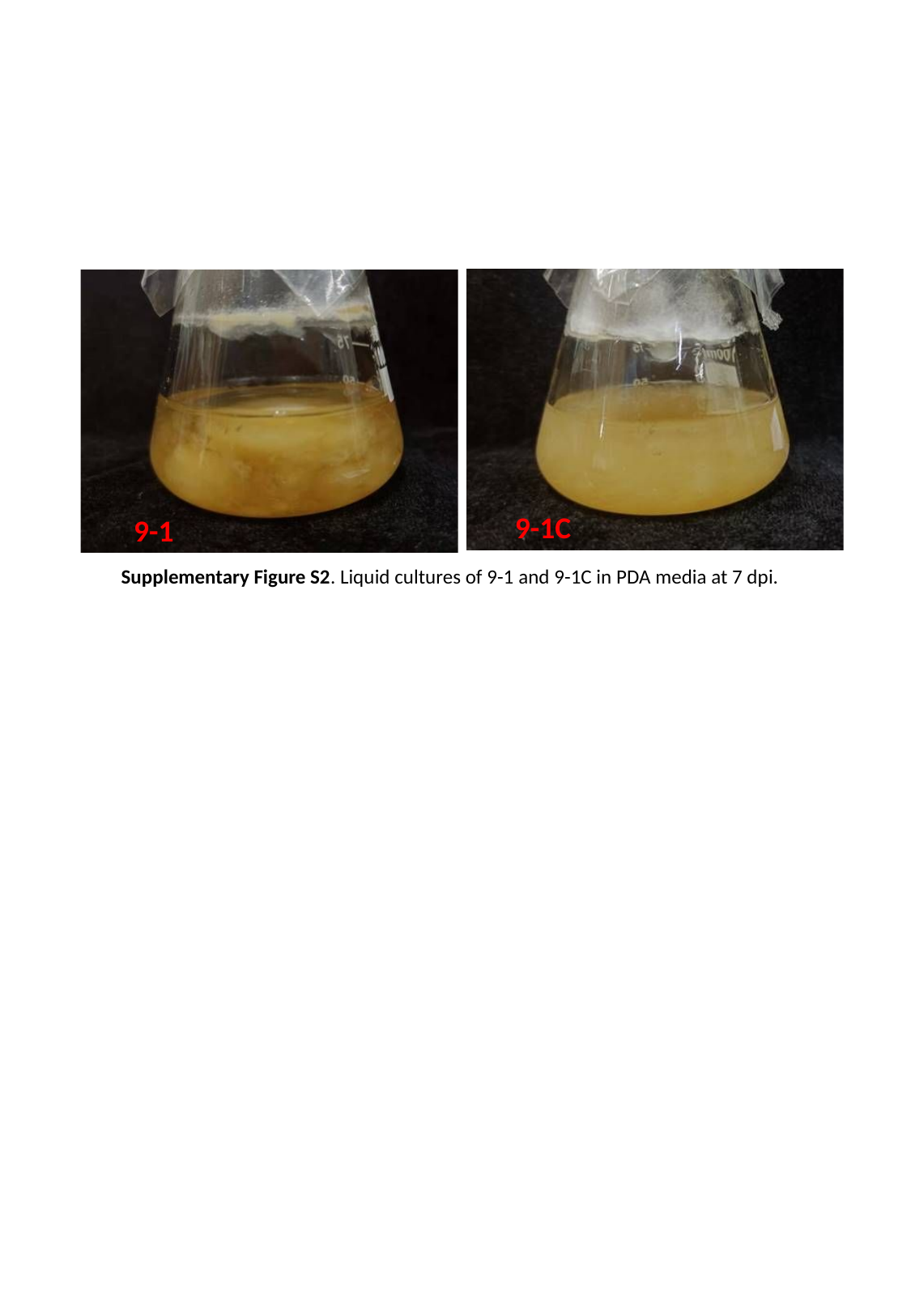

9-1C
9-1
Supplementary Figure S2. Liquid cultures of 9-1 and 9-1C in PDA media at 7 dpi.
